# Neglected implications of land-use and land-cover changes on the climate-health nexus

**DOI:** 10.1088/1748-9326/acd799

**Published:** 2023-06-02

**Authors:** Anton Orlov, Kristin Aunan, Malcolm N Mistry, Quentin Lejeune, Julia Pongratz, Wim Thiery, Antonio Gasparrini, Eilif Ursin Reed, Carl-Friedrich Schleussner

**Affiliations:** 1 CICERO Center for International Climate Research, Oslo, Norway; 2 Department of Public Health, Environments and Society, London School of Hygiene & Tropical Medicine, London, United Kingdom; 3 Department of Economics, Ca’ Foscari University of Venice, Venice, Italy; 4 Climate Analytics, Berlin, Germany; 5 Department of Geography, Ludwig-Maximilians-University Munich, Munich, Germany; 6 Max Planck Institute for Meteorology, Hamburg, Germany; 7 Department of Hydrology and Hydraulic Engineering, Vrije Universiteit Brussel, Brussels, Belgium; 8 The Centre on Climate Change & Planetary Health, London School of Hygiene & Tropical Medicine, London, United Kingdom; 9 Centre for Statistical Methodology, London School of Hygiene & Tropical Medicine, London, United Kingdom

**Keywords:** land use and land cover, mortality, climate, human health

## Abstract

Climate change can substantially affect temperature-related mortality and morbidity, especially under high green-house gas emission pathways. Achieving the Paris Agreement goals require not only drastic reductions in fossil fuel-based emissions but also land-use and land-cover changes (LULCC), such as reforestation and afforestation. LULCC has been mainly analysed in the context of land-based mitigation and food security. However, growing scientific evidence shows that LULCC can also substantially alter climate through biogeophysical effects. Little is known about the consequential impacts on human health. LULCC-related impact research should broaden its scope by including the human health impacts. LULCC are relevant to several global agendas (i.e. Sustainable Development Goals). Thus, collaboration across research communities and stronger stakeholder engagement are required to address this knowledge gap.

## 1 Introduction

Growing scientific evidence shows that climate change will substantially affect temperature-related mortality and morbidity, especially under high greenhouse gas (GHG) emission pathways. Stringent GHG mitigation and additional adaptation measures are needed to reduce the adverse health impacts of global warming. At the same time, several emission reduction scenarios that aim to achieve the Paris Agreement rely on substantial land-use and land-cover changes (LULCC). Furthermore, sustainable land management is essential for realising other Sustainable Development Goals (SDGs), including zero hunger, no poverty, life on land, and health.

In this context, LULCC can serve multiple purposes: (i) to reduce GHG emissions through ecosystem conservation, (ii) to create CO_2_ sinks through reforestation, afforestation, or bioenergy with carbon capture and storage, and (iii) to fulfil the growing demand for food and fibre through sustainable irrigation. For many countries, land-based mitigation measures play an important role in reducing national GHG emissions, which are intended in nationally determined contributions. At the same time, deforestation and forest degradation, which is mainly driven by agricultural expansion, continued to increase over the last decades. For instance, Africa and South America show the highest annual rates of net forest loss (FAO and UNEP 2020). Furthermore, many low- and middle-income countries exhibit large productivity gaps of crops given the increasing demand for food, whereas a large potential for a sustainable irrigation expansion, especially in sub-Saharan Africa, Eastern Europe, and Central Asia, is still unrealised (Rosa *et al* 2020).

## 2 Biogeophysical effects on climate induced by LULCC

**Figure 1:**
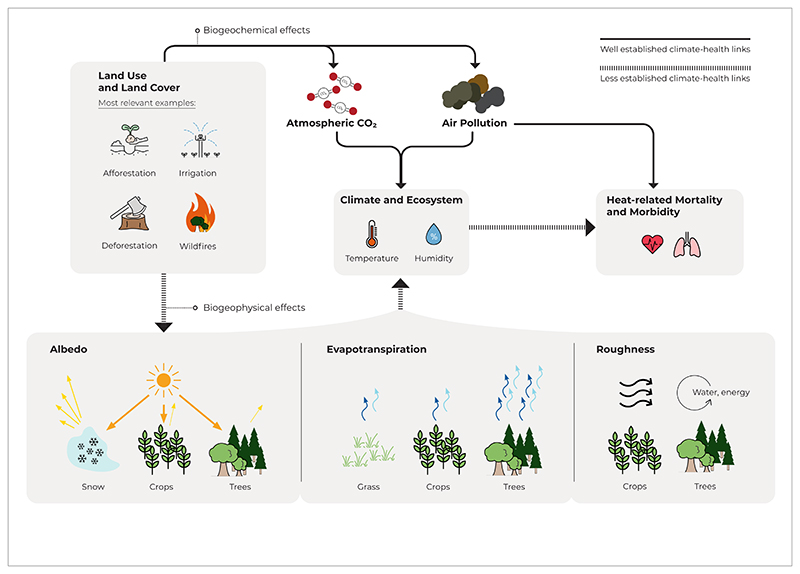
Climate-health implications of most relevant anthropogenic LULCC on health. A complete overview of LULCC can be found in Pongratz *et al* (2021). ‘Well established’ implies most studied.

**Figure 2:**
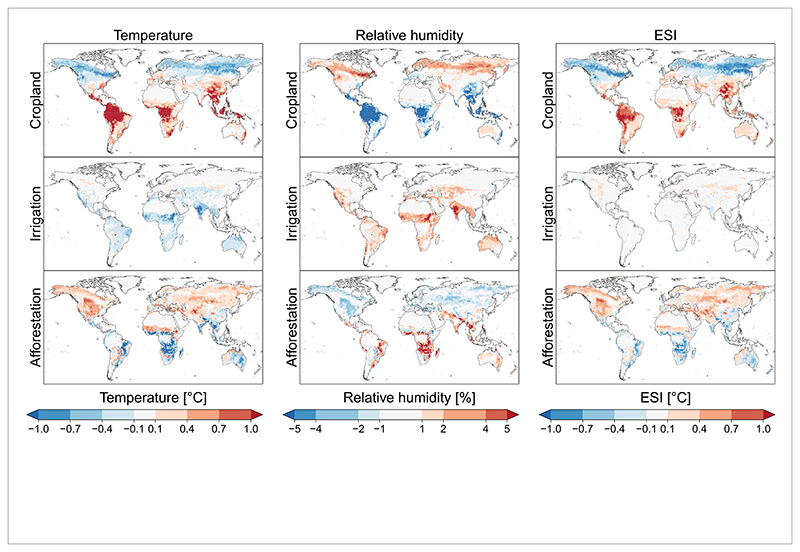
Absolute changes of annual average near-surface temperature, relative humidity, and environmental stress index (ESI) in response to the local effects of cropland expansion, irrigation expansion, and afforestation, which are simulated by the global climate model Community Earth System Model (CESM). ESI is a heat stress index, which is calculated using near-surface temperature, relative humidity, and solar radiation. ESI is found to be a relatively accurate substitute for wet bulb globe temperature (WBGT) index. Source: based data from Orlov *et al* (2023b).

LULCC have been mainly analysed in the context of land-based mitigation, local climate change, or water and food security. However, there is strong evidence that LULCC can substantially alter climate not only through the release of carbon to the atmosphere (biogeochemical effects) but also through changes in radiative, aerodynamic, and thermodynamic properties of the land surface (biogeophysical effects) (Bonan 2008) (figures 1 and 2). For instance, forest-cover changes (i.e. deforestation and afforestation) could change local temperatures by several degrees in some regions. Forests typically cool the surface in the tropics and midlatitudes, while high northern latitudes show a mixed signal (Winckler *et al* 2019). Furthermore, forests tend to dampen temperature extremes; for example, deforestation often leads to cooling in night-time (or winter) and warming in daytime (or summer), and vice versa for afforestation (Lee *et al* 2011, Duveiller *et al* 2018, Winckler *et al* 2019). Changes in land management, such as irrigation expansion, can also affect climate. Irrigation is found to substantially reduce exposure to temperature extremes in the past (Thiery *et al* 2020) but may increase moist heat (Mishra *et al* 2020). Thus, irrigation expansion will not only increase crop productivity but also could potentially affect health of rural population through biogeophysical effects on the local climate. Biogeophysical effects arising from LULCC can not only alter the local climate (local effects) but can also change climate in other regions through changes in atmospheric circulation (non-local effects) (Pongratz *et al* 2021, De Hertog *et al* 2023). For example, it has been found that irrigation-based agriculture in Asia could affect temperature and precipitation in Eastern Africa (de Vrese *et al* 2016).

While LULCC can substantially alter climate through local and non-local biogeophysical effects, little is known about the consequential health and socioeconomic responses. For instance, only a few studies investigate deforestation-induced impacts on productivity and capacity of labour (Alves de Oliveira *et al* 2021, Masuda *et al* 2021, Parsons *et al* 2021, Orlov *et al* 2023a). More importantly, to our best knowledge, even less research has been done on quantifying LULCC-induced impacts on health (Wolff *et al* 2021). LULCC changes could alter heat stress, thereby affecting the risk of heat-related morbidity and mortality. Wildfires enhance air pollution levels, while reforestation and afforestation can improve the air and water quality. Cardiovascular and respiratory diseases are major causes of death globally and their association with heat and air pollution is well established. However, a range of other comorbidities (e.g. kidney diseases) can also be important vulnerability factors for heat- and air pollution-related morbidity and mortality. In particular, rural population can potentially be affected to LULCC-induced changes in environmental stressors, because they live close to agricultural and forest areas. Globally, the share of rural population accounted for 43% in 2021. In developing countries, where significant LULCC might be needed for mitigation and food security purposes, the share of rural population is even higher than the global average. Moreover, rural populations tend to be relatively more vulnerable to adverse climate change impacts than urban, because rural communities often lack access to health care and have lower income.

Urban sprawl and urban planning also entail LULCC that can lead to impacts on health. The heat island effect makes urban areas especially exposed to heat stress compared to rural ones, due to the physical properties of the land cover types they are constituted of. On the other hand, specific land cover types within cities, primarily urban green spaces, are found to induce a locally cooling effect (Avashia *et al* 2021, Shah *et al* 2021). While health impacts from global climate change primarily driven by increasing GHG emissions are high on the research agenda and intensively studied, the LULCC-induced impact on human health is an important knowledge gap that needs to be addressed to effectively achieve SDGs, not the least for rural populations in heat-exposed regions. However, it should be noted that the health impacts of LULCC herein could have a significant adaptation potential if deployed in a climate-smart way maximising mitigation and adaptation benefits.

## 3 Methodological challenges

To understand how LULCC-induced climatic changes could affect health, several methodological and data limitations need to be addressed. Biogeophysical effects induced by LULCC are still uncertain and poorly understood (Perugini *et al* 2017). Current-generation Earth system models do not agree well on the space, magnitude, and even the sign of climatic responses to LULCC (Pongratz *et al* 2021, De Hertog *et al* 2023). Furthermore, it is very uncertain to which spatial scale LULCC-induced effects could expand (Crompton *et al* 2021). Regarding the uncertainty of biogeophysical effects, albedo- and roughness-related effects are relatively well understood in terms of sign, although the magnitude of effects is uncertain. Mostly, the change in evapotranspiration in response to LULCC is uncertain in terms of sign and magnitude. Moreover, most of existing global LULCC simulations are conducted as sensitivity experiments under idealised settings. More realistic and policy relevant LULCC simulations are needed.

Establishing the climate-health exposure-response functions (ERFs) should consider several methodological and data availability issues. The most comprehensive time-series dataset on daily mortality, which is collected by the Multi-Country Multi-City (MCC) collaborative research network, includes data predominantly at city levels. Yet, the potential climate impacts on health induced by LULCC, which is predicted to occur as part of future global land-use trends, could be more relevant for rural population. Moreover, many highly populated and heat-exposed countries (e.g. African countries) are still missing in the MCC dataset due to the lack of reliable data records. Most studies of temperature–mortality ERFs use near-surface daily mean temperature as the explanatory climatic exposure variable. However, forest-cover changes can have an impact on the diurnal cycle (night-time vs. daytime temperature responses), and LULCC could also affect humidity in a non-linear manner. For instance, the irrigation-induced impact on humidity could even result in an increase in moist heat, thereby potentially increasing human heat stress (Mishra *et al* 2020). Thus, for LULCC-related analyses, using the state-of-the art ERFs that are typically derived using 2 m air temperature could still lead to biased results.

While data on air temperature from *in situ* observations are spatially limited, land surface temperature (LST) data have a richer spatial and temporal coverage. To provide more accurate estimates of the temperature–mortality relationship, using LST in combination with humidity data instead of using ground stations observations could be an avenue for future research. However, this would require improving the quantification of relationships between air temperature and LST. It should also be noted that climate scientists investigate primarily surface-climate responses, while epidemiological ERFs are based on near-surface climate variables. Definitions of near-surface temperature are quite inconsistent across assessments, so the magnitude of surface and near-surface responses could substantially differ.

## 4 Outlook

To address these limitations, collaboration across research communities (i.e. climate science, ecology, meteorology, economics, and health), enhanced focus on rural areas, and last but not the least, stronger stakeholder engagement and field research are required. A better understanding of biogeophysical effects of LULCCC could help to assess the most suitable nature-based solutions that can address the objectives of mitigation, adaptation and achieving several SDGs. Improving the knowledge and strengthening the evidence for local co-benefits of protecting forests for adaptation and multiple SDGs can provide more incentives to effectively achieve forest protection. Regarding climate justice, this can be even more relevant for most vulnerable countries, which are most exposed to both impacts from global climate change and LULCC. Including the biogeochemical and biogeophysical effects of LULCC in impact assessments could allow to better address potential co-benefits or trade-offs across multiple SDGs.

## Data Availability

The data that support the findings of this study are openly available at the following URL/DOI: www.wdc-climate.de/ui/entry?acronym=DKRZ_LTA_1147_ds00002.
